# Showing NAFLD, as a key connector disease between Alzheimer’s disease and diabetes via analysis of systems biology 

**Published:** 2020

**Authors:** Elham Gholizadeh, Ali Khaleghian, Diba Najafgholi Seyfi, Reza Karbalaei

**Affiliations:** 1 *Department of Biochemistry, Semnan University of Medical Sciences, Semnan, Iran*; 2 *Gastroenterology and Liver Diseases Research Center, Research Institute for Gastroenterology and Liver Diseases, Shahid Beheshti University of Medical Sciences, Tehran, Iran*; 3 *Proteomics Research Center, Faculty of Paramedical Sciences, Shahid Beheshti University of Medical Sciences, Tehran, Iran *

**Keywords:** NAFLD, Alzheimer's disease, Diabetes mellitus, Type II, Male infertility, Bioinformatics

## Abstract

**Aim:**

This study was designed to perform network analysis of Alzheimers҆ disease and diabetes and to find their correlation with each other and other diseases/pathways.

**Background:**

Alzheimer’s disease (AD) as a neurodegenerative disease and diabetes as a metabolic disease are two major health problems in the recent years. The recent studies have reported their correlation and same spreading pathways of these two diseases together, but details of this relation are not well known yet at molecular level..

**Methods:**

In thermal proteome profiling (TPP) technique, after treatment of the extracted proteins by heat and drug concentration, the resulting proteins were analyzed by mass spectrometry. Enrichment analysis of these proteins led to development of AD and diabetes. First, corresponding genes for each disease were extracted from DisGeNET database and then, protein-protein interaction network was constructed for each of them using the search tool for retrieval of interacting genes and proteins (STRING). After analyzing these networks, hub-bottleneck nodes of networks were evaluated. Also, common nodes between two networks were extracted and used for further analysis.

**Results:**

High correlation was found between AD and diabetes based on the existence of 40 common genes. Results of analyses revealed 14 genes in AD and 12 genes in diabetes as hub-bottleneck 7 of which were common including caspase 3 (CASP3), insulin-like growth factor 1 (IGF1), catalase (CAT), tumor necrosis factor (TNF), leptin (LEP), vascular endothelial growth factor A (VEGFA), and interleukin 6 ( IL-6).

**Conclusion:**

Our results revealed a direct correlation between AD and diabetes and also a correlation between these two diseases and non-alcoholic fatty liver disease (NAFLD), suggesting that a small change in each of these three diseases can lead to development of any other diseases in the patients. Also, the enrichments exhibited the existence of common pathways between AD, diabetes, NAFLD, and male infertility.

## Introduction

 Recently, the correlation between different diseases and the risk factors that may cause various diseases have become a controversial issue. Thus, development of a reliable technique for detecting protein-protein interaction can be a big revolution in this field. Alzheimer’s disease (AD) as a neurodegenerative disease has been one of the major health problems in the recent years. The Alzheimer’s disease International (ADI) federation has reported that at least 46.8 million people have been affected by dementia and this figure has been anticipated to increase by 74.7 million by 2030 and 131.5 million by 2050, respectively (1). AD is thought to develop 20 years or more before manifestation of the symptoms (2-4), along with small changes in the brain that are unnoticeable to the affected person. The first symptoms of this disease are memory loss and language problems, occurring as a result of destroying or damaging nerve cells (neurons) in parts of the brain involved in thinking, learning ,and memory (cognitive function) (5). It has been shown that besides age and heredity, lifestyle is an important effector in progression of AD (6).

On the other hand, lifestyle has major effects on development of some other diseases, i.e., obesity, fatty liver, and diabetes (7). Diabetes mellitus is a heterogeneous metabolic disorder characterized by the presence of hyperglycemia due to deterioration of insulin secretion, defective insulin action, or both. Majority of diabetes cases are divided into two categories, type 1 and type 2. There are some cases ,which are difficult to be classified ,such as gestational diabetes mellitus (GDM), genetic mutations, diseases of the exocrine pancreas (such as cystic fibrosis) ,and other diseases or drug exposure (such as glucocorticoids, medications for treatment of human immunodeficiency virus(HIV)/ acquired immunodeficiency syndrome(AIDS), and atypical antipsychotics) (8). 

Many studies have been conducted on insulin signaling in the brain during normal adulthood and aging and in the individuals with AD. But, there is limited information on molecular correlation between AD and diabetes. However, several studies have established the direct effect of diabetes on AD (9-11). Much information is available regarding biology of each of these diseases separately, and there is an increasing interest for recognizing their pathophysiological intersection (12). In our previous study, both of these diseases were enriched by identifying the targets of Celecoxib using thermal proteome profiling (TPP). As a recently introduced proteomics technology, TPP demonstrates the potential for proteome profiling for large-scale analysis of proteome-ligand interactions including endogenous ligands, such as cofactors or metabolites, and other protein modifications. TPP facilitates identification of markers for drug efficacy and toxicity and provides an unbiased measure of drug-target engagement. As a mass spectrometry-based technique, TPP provides a rationale for adverse clinical observations and suggests repurposing of the drug for treatment of other diseases (13, 14).

Considering accuracy of TPP technique and detecting AD and diabetes in our previous study, herein it was attempted to analyze the correlation between these two widespread diseases. 

## Methods

The hippocampi of 5 male Rattus norvegicus, weighing 200 +/-10 g were separated and homogenized in radioimmunoprecipitation assay (RIPA) buffer and finally, were centrifuged in 20,000 g for 20 min at 4°C. The supernatant containing proteins was carefully separated from precipitates and concentration of protein solution was determined by Bradford assay. Then, the TPP procedure was done using Celecoxib concentrations of 20, 10, 5, 1, and 0.1µM, based on the protocols of TPP (13-16). Finally, the solarized proteins were extracted and fractionated by NanoDrop device. The proteins were identified by mass spectrometry-based laboratory protocol (17, 18). Following identification of proteins using the existing databases, enrichment analysis was used to determine the role of the protein set in different diseases using the Enrichr web tool (https://amp.pharm.mssm.edu/Enrichr/). This online database contains many different libraries, and the effect of genes on pathways, disease, and phenotypes is determined by entering the list of genes (ref Enrichr). The effects of the identified proteins on different processes were determined using this database. Considering high P-value of AD and diabetes, the correlation between these two diseases was investigated in the DisGeNET database. DisGeNET is a comprehensive discovery database providing information on the association of genes and variants with human diseases (19). The related genes of AD and diabetes were extracted from the DisGeNET platform and were used for further analysis. PPI network of each disease was constructed by the search tool for retrieval of interacting genes/proteins (STRING). STRING is a discovery platform, predicting protein-protein interactions (20). The resulting networks were imported in Cytoscape software, and the ClusterONE algorithm identified their topology properties as a plugin of this software (21). The relation between common genes and pathway enrichment was accomplished using ClueGO and CluePedia plugins of Cytoscape software (22, 23). An R package named as CINNA was used to find the best centrality method for selecting the most critical nodes (24, 25).

Every network contains nodes (such as genes or proteins) and edges/links (e.g., co-expression relationships or physical interactions) as their connections. Degree and betweenness are important centrality parameters in network biology that are useful for analyzing network topology. The term degree indicates edges/links of a node. Nodes with high degree values are called as hubs, and nodes achieving top-ten or top-five % of betweenness centrality are called as bottlenecks. So, hub-bottlenecks are nodes that are simultaneously hubs and bottlenecks. Average degree (A.D) and standard deviation (SD) of degrees were calculated, and nodes with a degree value above 2SD + A.D were selected as hub proteins. Also, the top 5% betweenness centrality measures were chosen as bottleneck proteins. Shared genes, hubs, and bottleneck proteins of these two networks were extracted and used for further analysis. In this study, Cytoscape software was used to analyze networks and extract hubs, hub-bottlenecks, and their first neighbors (26). 

## Results

**Figure 1:**
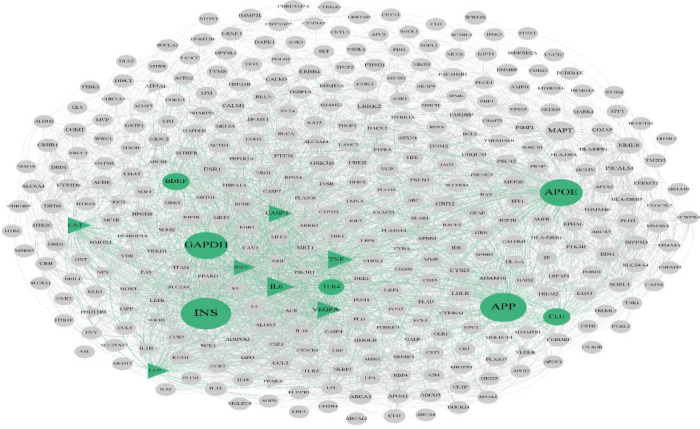
*PPI network for AD. This network includes 14 hub-bottlenecks (highlighted with green color) 7 of which are common with the identified hub-bottlenecks of diabetes, indicated with triangle*

**Figure 2:**
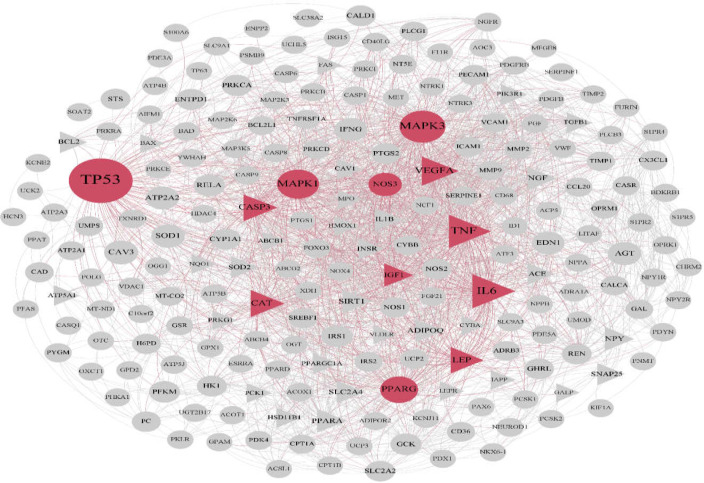
PPI network for diabetes. This network includes 12 hub-bottlenecks (highlighted with red color) 7 of which are common with the identified hub-bottlenecks of diabetes, indicated with triangle

**Figure 3:**
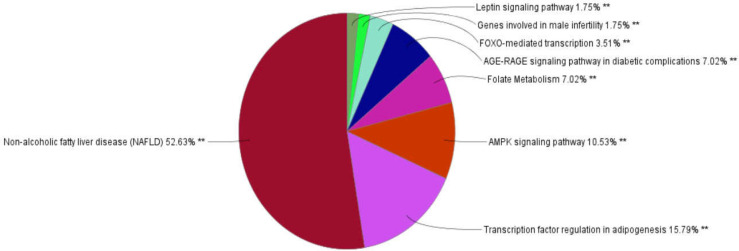
Pathways enriched by the common genes between AD and diabetes. AD and diabetes have the highest effect on NAFLD. It can be concluded that NAFLD is a key connector disease between these two diseases

After enrichment of the identified proteins from MS by Enrichr web tool, AD (Adj p-value: 0.00089) and diabetes (Adj p-value: 0.022) were identified interestingly. For detecting the correlation between AD and diabetes, the gene sets of these diseases were extracted from DisGeNET database. Based on this database, 386 and 523 related genes were extracted for AD and diabetes, respectively. Constructing PPI networks was done for each disease using STRING. AD network contained 347 nodes 14 of which were identified as hub-bottleneck (Figure 1 and Table 1). On the other hand, diabetes network included 211 nodes 12 of which were detected as hub-bottleneck in this network (Figure 2 and Table 1). Interestingly, 7 of these hub-bottlenecks were common between two networks including caspase 3(CASP3), insulin-like growth factor 1(IGF1), catalase (CAT), tumor necrosis factor (TNF), leptin (LEP), vascular endothelial growth factor A(VEGFA) ,and interleukin 6(IL-6). Also, 40 proteins were shared proteins between networks; their roles were analyzed via the ClueGO plugin (Table 2, Figure 3). Enrichment analysis showed 34 pathways in overall 15 of which were from the Kyoto encyclopedia of genes and genomes (KEGG) database, 5 of which were enriched via Reactome database and 14 pathways were identified by the WikiPathways database.

**Table 1:** Enriched hub-bottlenecks in AD and diabetes. Similar hub-bottlenecks are shown by asterisk

Disease	Name	Degree	Betweenness centrality
AD	CASP3*	84	0.017712
AD	BDNF	84	0.022581
AD	GAPDH	129	0.074805
AD	APP	140	0.085575
AD	IGF1*	76	0.009626
AD	INS	153	0.099242
AD	CAT*	72	0.016219
AD	LEP*	73	0.01045
AD	TNF*	104	0.025062
AD	IL-6*	121	0.032354
AD	APOE	135	0.074739
AD	VEGFA*	90	0.020626
AD	TLR4	74	0.016169
AD	CLU	76	0.022463
Diabetes	TNF*	103	0.050838
Diabetes	TP53	102	0.094941
Diabetes	CASP3*	84	0.027162
Diabetes	PPARG	75	0.029648
Diabetes	CAT*	73	0.027995
Diabetes	LEP*	75	0.025842
Diabetes	IGF1*	75	0.014226
Diabetes	NOS3	71	0.018537
Diabetes	VEGFA*	87	0.036401
Diabetes	MAPK1	85	0.03988
Diabetes	MAPK3	101	0.049828
Diabetes	IL-6*	101	0.052281

## Discussion

PPI network analysis and pathway enrichment as systems biology methods have been used to discover main proteins and pathways underlying complex diseases (27). During this blind study of systems biology, interestingly, AD and diabetes were found in the enriched diseases simultaneously, while the studied tissue was hippocampus. Some important common genes were found by extracting the genes related to these diseases, which were also effective in NAFLD, indicating high correlation between these three diseases. 

Many different signaling pathways were detected in this research (Table 1, Figures 1&2). In various studies, FOXO3 has been reported as a susceptible gene for human longevity; by aging or its expression is decreased in AD (28). 

**Table 2:** *Enriched pathways by the shared proteins between AD and diabetes. The results were extracted from 3 databases: KEGG, WikiPathways, and Reactome databases*

Ontology source	GOTerm	Adjusted P-value	Associated genes
KEGG	Adipocytokine signaling pathway	1.13778E-11	ADIPOQ, LEP, LEPR, NPY, PCK1, PPARA, PPARGC1A, SLC2A4, TNF
	Advanced glycation end products(AGE)-receptor of AGE(RAGE) signaling pathway in diabetes complications	7.53638E-12	BAX, BCL2, CASP3, IL-1B, IL-6, NOS3, PIK3R1, TGFB1, TNF, VEGFA
	AMP-activated protein kinase (AMPK) signaling pathway	1.11028E-12	ADIPOQ, IGF1, INSR, LEP, LEPR, PCK1, PIK3R1, PPARG, PPARGC1A, SIRT1, SLC2A4
	Amyotrophic lateral sclerosis (ALS)	5.99784E-06	BAX, BCL2, CASP3, CAT, TNF
	Apoptosis	1.78131E-06	BAX, BCL2, CASP3, FAS, NGF, PIK3R1, TNF
	Fluid shear stress and atherosclerosis	1.94221E-10	BCL2, CAV1, CYBA, HMOX1, IL-1B, NOS3, NQO1, PIK3R1, TNF, VEGFA
	FoxO signaling pathway	1.09653E-10	CAT, IGF1, IL-6, INSR, PCK1, PIK3R1, SIRT1, SLC2A4, SOD2, TGFB1
	Hypoxia-inducible factor 1(HIF-1) signaling pathway	2.35735E-08	BCL2, HMOX1, IGF1, IL-6, INSR, NOS3, PIK3R1, VEGFA
	Hypertrophic cardiomyopathy (HCM)	2.93532E-05	ACE, IGF1, IL-6, TGFB1, TNF
	Insulin resistance	6.54836E-10	IL-6, INSR, NOS3, PCK1, PIK3R1, PPARA, PPARGC1A, SLC2A4, TNF
	Longevity regulating pathway	2.31646E-12	ADIPOQ, BAX, CAT, IGF1, INSR, PIK3R1, PPARG, PPARGC1A, SIRT1, SOD2
	Non-alcoholic fatty liver disease (NAFLD)	7.09E-15	ADIPOQ, BAX, CASP3, FAS, IL1B, IL-6, INSR, LEP, LEPR, PIK3R1, PPARA, TGFB1, TNF
	p53 signaling pathway	1.3591E-05	BAX, BCL2, CASP3, FAS, IGF1
	TNF signaling pathway	6.98289E-06	CASP3, FAS, IL-1B, IL-6, PIK3R1, TNF
	Type II diabetes mellitus	2.81374E-06	ADIPOQ, INSR, PIK3R1, SLC2A4, TNF
Reactome	FOXO-mediated transcription	2.09583E-08	CAT, CAV1, NPY, PCK1, PPARGC1A, SIRT1, SOD2
	FOXO-mediated transcription of oxidative stress, metabolic and neuronal genes	4.15153E-07	CAT, NPY, PCK1, PPARGC1A, SOD2
	Interleukin 4(IL-4) and interleukin 13(IL-13) signaling	2.25725E-08	BCL2, HMOX1, IL-1B, IL-6, PIK3R1, TGFB1, TNF, VEGFA
	Transcriptional regulation of white adipocyte differentiation	6.99501E-11	ADIPOQ, LEP, PCK1, PPARA, PPARG, PPARGC1A, SLC2A4, TGFB1, TNF
	VEGFA-VEGF receptor 2(VEGFR2) pathway	9.37356E-06	CAV1, CYBA, NOS3, PIK3R1, VEGFA
WikiPathways	Adipogenesis	6.4079E-14	ADIPOQ, FAS, IGF1, IL-6, LEP, PCK1, PPARA, PPARG, PPARGC1A, SLC2A4, TGFB1, TNF
	AMPK signaling	8.09075E-07	ADIPOQ, INSR, LEP, LEPR, PIK3R1, SLC2A4
	ALS	1.34735E-06	BAX, BCL2, CASP3, CAT, TNF
	Apoptosis	1.32024E-07	BAX, BCL2, CASP3, FAS, IGF1, PIK3R1, TNF
	Folate metabolism	4.39784E-08	CAT, IL-1B, IL-6, INSR, MPO, SOD2, TNF
	Genes involved in male infertility	1.61695E-08	ABCB1, BCL2, CAT, FAS, INSR, NOS3, NQO1, SOD2, TNF
	Hepatitis B infection	2.8804E-07	BAX, BCL2, CASP3, FAS, IL-6, PIK3R1, TGFB1, TNF
	Leptin signaling pathway	1.30077E-06	BAX, IL-1B, LEP, LEPR, NOS3, PIK3R1
	NAFLD	1.7843E-14	ADIPOQ, BAX, CASP3, FAS, IL-1B, IL-6, INSR, LEP, LEPR, PIK3R1, PPARA, TGFB1, TNF
	Photodynamic therapy-induced activator protein 1(AP-1) survival signaling	4.08953E-06	BAX, BCL2, FAS, IL-6, TNF
	T-Cell antigen receptor (TCR) signaling pathway	2.47953E-05	FAS, IL-1B, IL-6, PIK3R1, TGFB1
	TNF alpha signaling pathway	1.45408E-05	BAX, CASP3, CYBA, IL-6, TNF
	Transcription factor regulation in adipogenesis	3.4134E-14	ADIPOQ, IL-6, INSR, LEP, PPARG, PPARGC1A, SLC2A4, TNF
	Vitamin B12 metabolism	2.19331E-07	IL-1B, IL-6, INSR, MPO, SOD2, TNF

Activation of FOXO3 initially acts as a neuroprotective agent; however, later on it plays a role in promoting cell death by upregulating Bim and FasL. Decreasing expression of FOXO3 might be an effective way to prevent or delay irreversible process of neurodegeneration (28). IGF1 as another common known pathway has a similar function to insulin and stimulates glucose transport into the cell (29). It has been reported that IGF1 can influence pathogenesis of AD through regulation of α-/β-secretase activity (30). 

IGF1 can influence Aβ clearance from the brain by promoting Aβ transport over the blood-brain barrier, unbalanced β-amyloid, occurring in diabetes is associated with neurite degeneration and neuronal loss (30), highlighting the correlation between AD and diabetes. The other important pathways are TNF and CASP3; both causing cell death. TNF expression is increased in acute and chronic systemic inflammation and there is a direct relation between TNF and CASP3; on the other hand, an increase in TNF expression causes an increase in CASP3 expression (31). An increase in TNF expression causes subsequent cognitive decline and long-term cognitive impairment (32) also causes insulin resistance and diabetes (33). As a key regulator of energy balance, leptin, the other important enriched pathway has been reported to be a helpful gene in AD and diabetes. Pathways activated by leptin in the brain have neuroprotective roles (34) also, leptin may be a potentially useful adjunct to insulin treatment in management of diabetes (35). IL-6 regulates acute-phase responses of cytokines and lymphocyte stimulatory factors. IL-6 has been reported to have two differential roles in modulating insulin sensitivity, an enhancer and an inhibitor of insulin action (36). Increased level of IL-6 is a sign of pathological events including neuroinflammation and neurodegeneration (37). In conclusion, inhibition of IL-6 causes a rationale strategy for targeting onset or further progression of AD (38). Vascular endothelial growth factors (VEGF) are a subfamily of growth factors also enriched in this research and acting as signaling proteins for vasculogenesis and angiogenesis (39). The recent studies have proved that in diabetes, angiogenesis is decreased by inhibition of VEGF through corticosteroids, so the use of anti-VEGF agents is proposed for management of diabetes complications (40). Vascular and AD pathologies are related and the patients with AD carry more functional promoter genes for VEGF, resulting in the elevated levels of VEGF (41). Considering these studies, VEGF have major role in AD as well as diabetes. All these mentioned common genes have a huge effect on both AD and diabetes and small changes in them can influence situation of the patients.

The results of pathway enrichment analysis on common genes between AD and diabetes showed a strong correlation between AD, diabetes, and NAFLD (Fig.2). A close association between AD and NAFLD and diabetes and NAFLD has been proven in the recent studies (42, 43). It is well known that liver has fundamental importance in regulation of metabolism and insulin sensitivity, as well as diabetes (44, 45). Liver dysfunction causes diabetes, on the other hand, there is a direct correlation between diabetes and NAFLD, and as a result, NAFLD is an important risk factor for diabetes (46). Recently, studies have suggested that diabetes could be related to an increased risk for AD (47, 48) still, there is no clear evidence about causative relationship between diabetes and cognitive decline in the patients with AD. (11). Thus, it can be proposed that NAFLD is a key connector disease between AD and diabetes. 

The other important pathway is related to the genes involved in male infertility by involvement of 8 common genes between them. Since, glucose metabolism is important for supplying basic cell activity, as well as specific functions, such as motility and fertilization ability in mature sperm, it is also considered as an important event in spermatogenesis (49, 50). Based on the recent evidence, diabetes has a destructive effect on male fertility through influencing sperm motility, sperms҆ DNA integrity, and ingredients of seminal plasma (51, 52). The correlation between AD and male fertility has been proved by the effect of amyloid precursor protein in both diseases, although function of this protein in male fertility is a novel subject and needs more investigations (53). NAFLD is strongly associated with severity of germinal epithelial damage. Additionally, the testis has been identified as a probable target organ for damage caused by NAFLD, suggesting that NAFLD can influence fertility in males through damaging testis (54). According to the previous studies, there is a correlation between AD, NAFLD, and diabetes; also in this study, these diseases were enriched with high possibility.

Altogether, in this research, firstly, a direct correlation was found between AD and diabetes. Following further analysis, connecting function of NAFLD on these two diseases was revealed, which based on the current epidemiologic studies, there was a causative relation between these diseases. Also, the enrichments exhibited the genes involved in male infertility, suggesting the existence of common pathways between AD, diabetes, NAFLD, and male infertility. Clearly, more extensive experimental and clinical studies are needed, in order to clarify molecular correlations between these diseases
